# Light-Guided Cyborg Beetles: An Analysis of the Phototactic Behavior and Steering Control of *Endebius florensis* (Coleoptera: Scarabaeidae)

**DOI:** 10.3390/biomimetics10080513

**Published:** 2025-08-06

**Authors:** Tian-Hao Zhang, Zheng-Zhong Huang, Lei Jiang, Shen-Zhen Lv, Wen-Tao Zhu, Chao-Fan Zhang, Yi-Shi Shi, Si-Qin Ge

**Affiliations:** 1 Key Laboratory of Animal Biodiversity Conservation and Integrated Pest Management, Institute of Zoology, Chinese Academy of Sciences, Beijing 100101, China; shanezth@126.com (T.-H.Z.); huangzz@ioz.ac.cn (Z.-Z.H.); bugarmy@foxmail.com (L.J.); 2 University of Chinese Academy of Sciences, Beijing 100049, China; 3 College of Computing and Data Science, Nanyang Technological University, Singapore 639798, Singapore; shenzhen.lyu@ntu.edu.sg; 4 Anhui Institute of Optics and Fine Mechanics, Hefei Institutes of Physical Science, Chinese Academy of Sciences, Hefei 230031, China; wentao.zhu@nyu.edu (W.-T.Z.); zcfan@aiofm.ac.cn (C.-F.Z.); 5 Science Island Branch, Graduate School of USTC, Hefei 230026, China; 6 Center for Materials Science and Optoelectronics Engineering, University of Chinese Academy of Sciences, Beijing 100049, China; 7 Aerospace Information Research Institute, Chinese Academy of Sciences, Beijing 100094, China

**Keywords:** cyborg beetle, insect–machine hybrid, phototaxis, light-guided, steering control

## Abstract

Cyborg insects offer a biologically powered solution for locomotion control, but conventional methods typically rely on invasive electrical stimulation. Here, we introduce a noninvasive, phototaxis-based strategy to steer walking *Endebius florensis* beetles using light-emitting diode (LED) stimuli. Electroretinogram recordings revealed spectral sensitivity to blue, green, and yellow light, with reduced response to red. Behavioral assays demonstrated robust positive phototaxis to blue light and negative phototaxis to yellow. Using these findings, we built a wireless microcontroller-based backpack emitting directional blue light to induce steering. The beetles reliably turned toward the activated light, achieving angular deflections over 60° within seconds. This approach enables repeatable, trauma-free insect control and establishes a new paradigm for biohybrid locomotion systems.

## 1. Introduction

Cyborg insects represent a hybrid technique that integrates an artificial system onto a living insect. This system is grounded in neurobiology, allowing for artificial intervention in the insect’s neural pathways. By leveraging the insects’ own energy, environmental sensing abilities, and locomotor capabilities, behaviors such as walking, flying, and jumping can be controlled. This enables precise movement manipulation. Compared to purely mechanical robots, especially those on a smaller scale, cyborg insects require less energy. Their movements are powered by the insect’s biological processes, with artificial systems only providing stimulation to induce movement and control, without the need for complex mechanical structures. As a result, cyborg insects demonstrate superior obstacle navigation, robustness, and endurance.

Since Holzer and Shimoyama’s successful artificial control of cockroach locomotion in 1997 [1], numerous studies have explored various motion control strategies for cyborg insects. These studies have examined factors like electrical stimulation waveform [1,2,3], timing [4,5], stimulation sites and organs [3], and locomotive behaviors [3,4,5]. Almost all control schemes are realized using electrical pulses as the stimulation source. Electrical pulses are typically used for stimulation, requiring the stimulation interface to penetrate the exoskeleton for implantation within the insect; however, this method is invasive, as it requires penetrating the insect’s exoskeleton to implant the interface, which can cause damage. Non-invasive alternatives include early-metamorphosis insertion technology (EMIT) [6,7] and conformal electrodes [8], but these methods are cumbersome and require specialized training. EMIT also has limitations, such as requiring the insect to undergo holometabolous metamorphosis, which restricts its use. These factors pose challenges for the widespread application of cyborg insects.

Phototaxis, the natural tendency of insects to move toward or away from light, is an important behavioral trait influencing various life activities, such as feeding, mating, reproduction, and predator avoidance. Insects moving toward light exhibit preferences for specific wavelengths of light. For example, the cigarette beetle *Lasioderma serricorne* prefers light at wavelengths of 375 nm and 470 nm [9], while mosquitoes like *Aedes infirmatus*, *Aedes vexans*, and *Culex nigripalpus* are attracted to light at 470 nm and 502 nm [10]. Currently, insect phototaxis is mainly applied in agricultural and sanitary pest control [11,12,13], but it has also been explored in relation to biological control, chemical attractants, and habitat management [14]. Light-emitting diodes (LEDs) have been increasingly used in place of traditional light sources like xenon, mercury, and fluorescent lamps due to their compact size, lightweight, pure emission wavelengths, and cost-effectiveness. Beyond their use in pest management, LEDs have great potential in the development of wearable insect devices. Studies have shown that LED lights can elicit a phototactic response from insects [15,16,17], with stable wavelength preferences [18,19], providing a foundation for utilizing light to direct insect movement.

The rhinoceros beetle *Endebius florensis* (Coleoptera: Scarabaeidae) is a large beetle species primarily distributed in Southeast Asian regions, including Flores Island, Sangeang Island, etc. Adults exhibit pronounced sexual dimorphism, with males possessing well-developed cephalic and thoracic horns (collectively referred to as the male phenotype), while females lack these morphological traits. Compared to other species within the genus, *E. florensis* is distinguished by the presence of denticles near the base of its thoracic horn, whereas its cephalic horn lacks such denticles [20]. This species is commonly reared as an ornamental insect, yet its biological characteristics and ecological significance remain poorly documented. Recent studies on the biomimetic applications of its hindwings have revealed its potential academic value [21]. Owing to the large body size, strong load-bearing capacity, distinct phototaxis, and commercial availability of *E. florensis*, we selected it as a carrier insect for our light-guided behavioral experiments.

The goal of this study is to explore methods for controlling insect locomotion using light attraction and assess the feasibility and potential applications of this approach. We conducted electrophysiological and behavioral tests on the compound eyes and phototactic responses of *E. florensis* to common visible-light LEDs. Based on these results, we selected two blue LEDs as the light source and mounted them on a wirelessly controlled microcontroller, forming a light-guided electronic backpack for insect locomotion control. This backpack was attached to the pronotum of the beetles, successfully inducing changes in directional walking through remote control.

This research not only demonstrates the feasibility of light-guided insect locomotion but also establishes a general framework for future studies. The light-guided cyborg beetle shows that optical signals can induce precise directional movement in insects, opening new possibilities for the design and application of cyborg insects.

## 2. Materials and Methods

### 2.1. Study Animals

The larvae of *E. florensis* were reared at a breeding base in Zhangzhou, Fujian Province, China. After eclosion, they were sent to Beijing and fed in the greenhouse of the Institute of Zoology, Chinese Academy of Sciences. All individuals were identified by Zheng-Zhong Huang, Institute of Zoology, Chinese Academy of Sciences. The rearing conditions were maintained at a temperature of 25 ± 3 °C, with a relative humidity of 70% ± 5%, and a photoperiod of 12L:12D. Each adult was individually reared in a 14.6 cm × 10.5 cm × 9.7 cm box, with the bottom lined with 4 to 5 cm of fermented wood shavings. Adults that had not emerged from dormancy were fed with jelly, and their dormancy status was confirmed daily by observing the consumption of the jelly. Active male and female adults that had emerged from dormancy were selected for the experiment.

### 2.2. ERG Measurement of Compound Eyes

**Figure 1:**
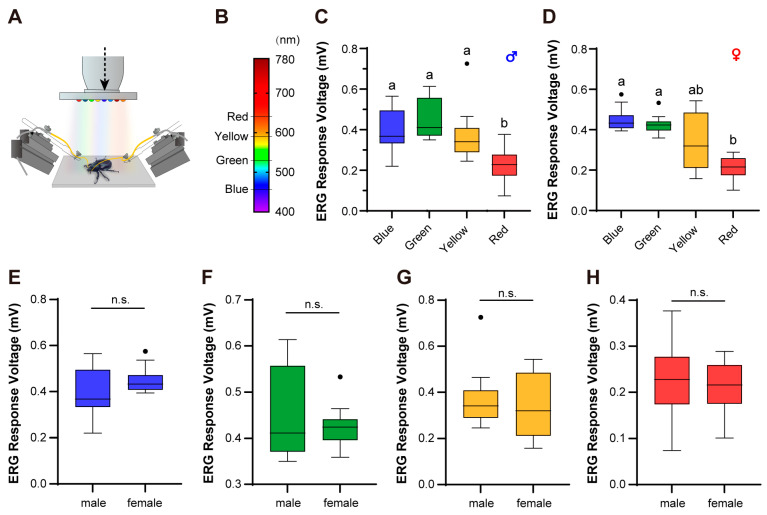
The electrophysiological responses of *E. florensis* compound eyes. (**A**) ERG equipment. (**B**) The spectral distribution of the test light source. (**C**,**D**) The quantification of the ERG voltage responses of male (**C**) and female (**D**) *E. florensis*. Different letters above the bars indicate a significant difference between ERG responses at the 0.05 level. (**E**–**H**) The quantification of the ERG voltage responses stimulated by blue (**E**), green (**F**), yellow (**G**), and red (**H**) light. n.s. indicates no significant difference between male and female individuals (*p* > 0.05).

To conveniently generate Electroretinogram (ERG)-stimulating light signals, we developed a microscope-matched light device capable of producing five colors of light (Figure 1A). This light source is equipped with blue (463–469 nm), green (516–525 nm), yellow (586–596 nm), red (625–645 nm), and white LEDs. Among them, the white LED serves as the illumination light for sample connection and observation, while the other light sources generate light stimuli of corresponding wavelengths. Additionally, the light device includes several 1 kΩ potentiometers (3296W-1-103LF, BOURNS, Riverside, CA, America), one microprocessor (ATMEGA 328P, Microchip, Chandler, AZ, America), one infrared receiver tube, and other external circuit components. The potentiometers are used to adjust the illuminance of the LEDs. The microprocessor is responsible for interpreting signals sent by the infrared remote control and activating the corresponding wavelength of the light source.

The light device is programmed with two control modes: regulation mode (constant-light mode) and test mode (timed mode). In test mode, after each command is received, the corresponding LED will turn on and then automatically turn off after a set period of time, ensuring a consistent duration of light stimulation. Conversely, in regulation mode, after each command is received, the corresponding LED will remain on until it receives another command to switch it off, which is typically used for illumination when fixing samples and for pre-testing.

The ERG experiments were conducted in a completely dark environment. The test individuals which had adapted to darkness for at least 1 h were decapitated. Subsequently, the freely movable head appendages such as antennae and labial palpi were removed. The heads were then fixed onto the experimental bench using Blu Tack, with the tested area of the compound eyes kept vertical. Both the recording electrode and the reference electrode were glass electrodes filled with conductive fluid (consisting of 128.34 mM NaCl, 4.69 mM KCl, and 1.89 mM CaCl_2_·2H_2_O in water). The tip of the recording electrode was placed on the surface of the compound eyes, while the reference electrode was attached to the exoskeleton of the head.

The illuminance of the light source at the focal length of the stereo microscope was pre-adjusted to 100 lux, and the stimulation duration was set to 5 s. The compound eyes were stimulated sequentially with blue (463–469 nm), green (516–525 nm), yellow (586–596 nm), and red (625–645 nm) light. There was an interval of 30 s between each stimulation, and each wavelength was stimulated three times. A total of six individuals participated in the test.

### 2.3. Phototactic Behavioral Test

**Figure 2:**
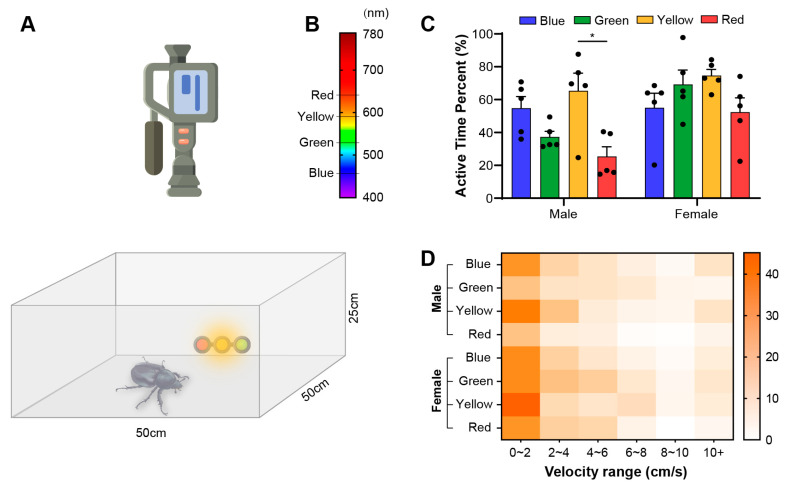
The behavioral expression of *E. florensis*. (**A**) Behavioral test chamber and video-capture equipment. The size of the chamber is 50 cm in both length and width, and 25 cm in height. (**B**) The spectral distribution of the test light source. (**C**) The active time of *E. florensis* as a percentage of total test time under different light attractions. * indicates a significant difference (*p* < 0.05). (**D**) Speed distribution under different light-induced conditions. Color blocks of different depths represent the percentage of time that an individual’s movement speed falls within the speed range, compared to the total time, under specific lighting conditions.

We constructed a 50 cm × 50 cm square chamber to test the behavioral responses of *E. florensis* towards different wavelengths (Figure 2A). The chamber was enclosed on all sides with transparent acrylic panels to prevent individuals from escaping, and a camera was mounted on top for vertical angle recording. LED light panels (10 cm × 10 cm) in blue (463–469 nm), green (516–525 nm), yellow (586–596 nm), and red (625–645 nm) were used as attractive light sources and placed at the center of one side of the chamber. The illuminance of the light sources was adjusted so that the illuminance at the starting point, 25 cm away from the light sources, was 200 lux.

The test individuals, which had been pre-adapted to darkness for at least 1 h, were placed at the starting position in the center of the chamber, with their body axes oriented perpendicularly to the direction of the light (i.e., one side of the individual was in the illuminated area, and the other side was in the dark area) (Figure 2A). The insects were allowed to move freely within the chamber, and their behavior was recorded using a digital video camera (DV).

### 2.4. Data Analysis

The ERG records were acquired using the LabScribe software (v.4.3.4.0), and the potential difference between the resting and the action potential was directly obtained using the software’s built-in tools. The recorded data were then statistically analyzed using PASW Statistics 18 software. A one-way ANOVA with Tukey’s method was conducted to analyze the electrophysiological signals under different light stimuli, while san independent-samples *t*-test was used to analyze the signals between different genders, and the significance of differences was assessed.

Behavioral videos were recorded using a digital video (DV) recorder and uniformly trimmed to the first 30 s of the test for analysis. The videos were shot at a frame rate of 30 frames per second (fps). During analysis, one frame was extracted every 10 frames to mark the position and orientation of the insects. Subsequently, the coordinates of the insects’ center of gravity (represented by the scutum) in each extracted frame were manually marked and exported as csv files. Frame extraction and manual coordinate marking were accomplished using a custom Python 3.11.2 script. Meanwhile, the software ImageJ 2.1.0 was used to calibrate the pixel-to-distance conversion ratio based on the chamber size (50 cm). Following this, the coordinate data were processed in Excel to calculate parameters such as movement distance, movement speed, angle distribution, distance from the light source, etc. Partial results were subjected to one-way ANOVA using PASW Statistics 18 software to assess the significance of differences. The method of analysis for the insect–machine hybrid test was similar to that of the behavioral analysis. The difference lay in the additional marking of head coordinates, which were used to calculate body-steering angles.

After completing the data analysis, the software Graphpad Prism 9.5.0 and custom Python scripts were used for plotting the data. The final layout and design of the figures were completed in Adobe Illustrator 19.

## 3. Results

### 3.1. Electroretinogram

We stimulated the compound eyes of the female and male *E. florensis* using four different wavelengths of light sources and recorded the ERG response signals. The results showed that the ERG signals generated by females under blue and green light stimulation were significantly higher than those under red light (*p* < 0.05). The ERG signals under yellow light stimulation exhibited a large variation range, with the mean value being lower than those under blue and green light but higher than that under red light. However, there were no statistically significant differences between yellow light and the other three wavelengths (*p* > 0.05) (Figure 1D). For male *E. florensis*, the ERG signals under blue, green, and yellow light stimulation were similar and not significantly different (*p* > 0.05), but all were significantly higher than those under red light (*p* < 0.05) (Figure 1C). Across all four wavelengths of stimulation, there were no significant differences in ERG signals between males and females (*p* > 0.05) (Figure 1E–H).

### 3.2. Behavioral Responses Under Different Wavelength

We recorded the behavior of *E. florensis* under different wavelengths of attractive light sources and analyzed their activities and phototaxis characteristics. The results showed that more than half of the time, males were in motion under blue and yellow light (54.83% ± 15.68% and 65.39% ± 23.97%, respectively), with a slightly lower proportion of motion time under green light (37.30% ± 7.68%). However, there were no significant differences between blue, green, and yellow light attractions. Under red light, the proportion of motion time was only 25.39% ± 13.26%, which was significantly lower than under yellow light (*p* < 0.05) but not significantly different from green and blue light (*p* > 0.05) (Figure 2C). In contrast, female *E. florensis* showed activity levels above 50% under all four light sources (55.06% ± 19.80%, 69.21% ± 19.54%, 74.61% ± 8.36%, and 52.36% ± 19.36%, respectively), with no significant differences (*p* > 0.05) (Figure 2C). Under blue and yellow light attractions, there were no significant differences in activity levels between males and females (blue light: *t* = −0.020, *p* = 0.985; yellow light: *t* = −0.812, *p* = 0.454; independent-samples *t*-test). However, under green and red light, female individuals showed significantly higher activity levels than males (green light: *t* = −3.399, *p* = 0.018; red light: *t* = −2.570, *p* = 0.037; independent-samples *t*-test). The speed-distribution heatmap showed that the movement speed of *E. florensis* was mainly between 0 and 2 cm/s, with a gradual decrease in subsequent speed intervals. During movement, individuals occasionally flew for a short distance, resulting in an increase in the proportion of time spent in the speed range above 10 cm/s (Figure 2C).

**Figure 3:**
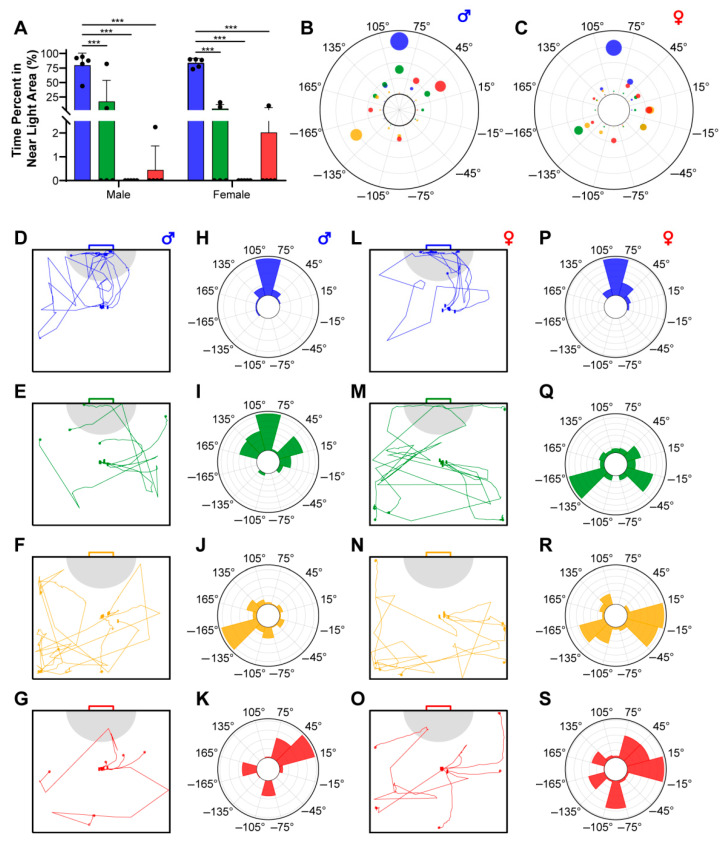
The phototactic behavior of *E. florensis*. (**A**) The percentage of time the individual remained in the near-light area (NLA). *** indicates an extremely significant difference (*p* < 0.001). (**B**,**C**) The quantification of the angle distribution of male (**B**) and female (**C**) *E. florensis*. (**D**–**G**) The movement trajectory of male *E. florensis* under blue (**D**), green (**E**), yellow (**F**), and red (**G**) lighting condition. The gray area represents the near-light area, which is a semicircle with a radius of 12.5 cm. (**H**–**K**) The angle distribution of males in the chamber under blue (**H**), green (**I**), yellow (**J**), and red (**K**) lighting conditions. The different bars represent the percentage of the total time that individuals remained within different angle ranges. (**L**–**O**) The movement trajectory of females under different lighting conditions. (**P**–**S**) The angle distribution of females in the chamber under different lighting conditions.

We define the semicircular area with a radius of 12.5 cm from the center of the light source as the near-light area (NLA, as illustrated by the gray regions in Figure 3D–G,L–O) and record the duration of time individuals spend within this area as a criteria for assessing their phototactic behavior. Figure 3A demonstrates that both female and male *E. florensis* exhibit higher proportions of NLA residence time under blue light compared to green, yellow, and red light attractions, with extremely significant differences (*p* < 0.001). Under green and red light attractions, some individuals move toward or remain in the NLA, whereas under yellow light, no individuals enter the NLA at all. There are no significant differences in NLA residence time between male and female individuals under green, yellow, and red light (*p* > 0.05).

Another criterion for determining an individual’s phototaxis is the distribution of the deviation angle of the body’s centroid position relative to the initial direction. In this study, the starting position was located at the center of the chamber, with the initial direction set at 0° and the light-source direction at 90°. The chamber was evenly divided into 12 sectors centered at the starting point, each spanning a 30° range. The initial direction (ID) was distributed within the −15° to 15° sector, and the light direction (LD) was distributed within the 75° to 105° sector. Figure 3D–S, respectively, showcase the activity trajectories and deviation-angle distributions of male and female *E. florensis* under different wavelengths. Specifically, both female and male individuals exhibited strong positive phototaxis under blue light, as evidenced by their rapid orientation towards the light source upon release (Figure 3D,L) and the high-frequency distribution of their activity trajectories in the light direction sector (Figure 3H,P). Under green light, only 2 out of 5 males passed through the NLA, and among the 2 individuals that eventually stayed in the LD sector, 1 did not enter the NLA (Figure 3E); among the 2 individuals that entered the NLA, 1 spent a relatively long time there (Figure 3A). Therefore, despite having the highest distribution in the LD sector illuminated by green light (Figure 3I), we do not consider male *E. florensis* to exhibit stable phototaxis towards this wavelength. Additionally, females under green light (Figure 3M,Q) and both sexes under yellow and red light (Figure 3F,G,J,K,N,O,R,S) did not exhibit obvious phototaxis. Notably, all individuals of both sexes tested under yellow light did not enter the NLA (Figure 3A,F,N), and their deviation-angle distributions were mainly oriented away from the light source (Figure 3J,R), suggesting a possible negative phototaxis towards yellow light.

### 3.3. Construct and Test of Light-Guided Cyborg Beetle

The wavelengths of LED light sources and the gender of individuals used were determined through electrophysiological and behavioral experiment results. Among these, ERG reveals the physiological mechanisms of the insect visual system, while behavioral studies demonstrate the actual behavioral responses of insects to specific light sources. The combination of both approaches enables a more comprehensive understanding and application of insect phototaxis.

**Figure 4:**
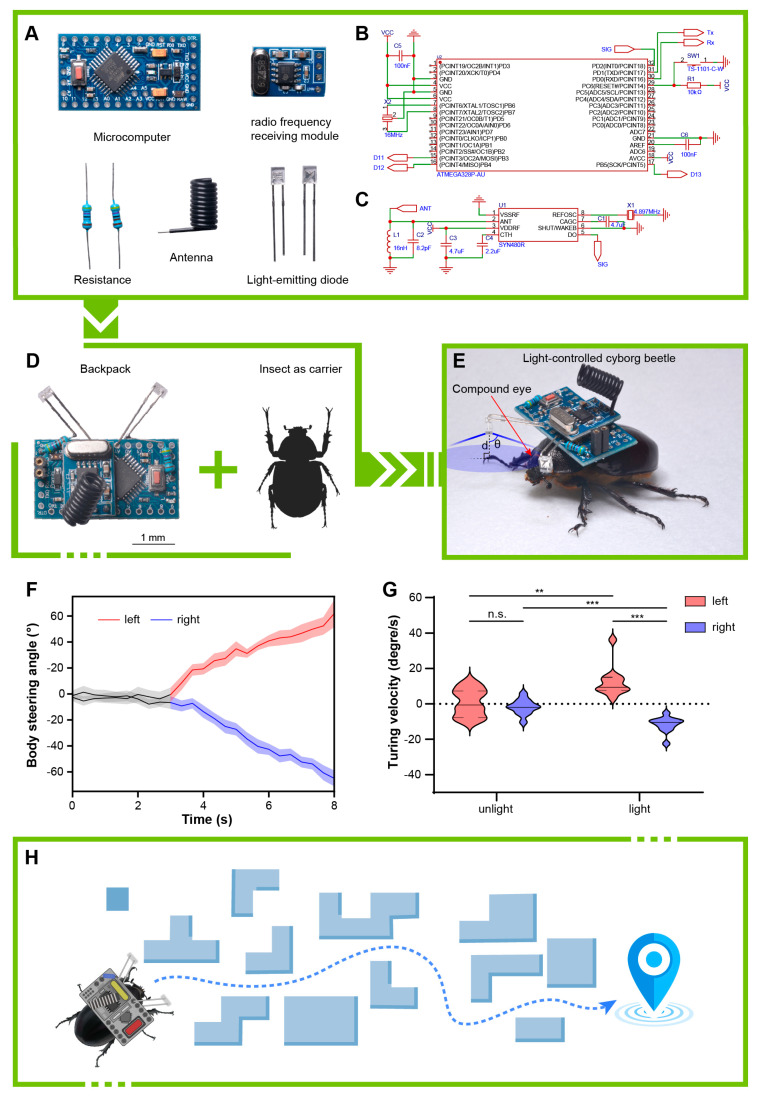
Remote control of the cyborg beetle. (**A**) The composition of the components of the electronic backpack. (**B**) A schematic diagram depicting the minimum system requirements of the ATMEGA 328P microcomputer unit. (**C**) A schematic diagram of the circuit for the RF-receiving module. (**D**) The assembled electronic backpack and the insect serving as carrier. The scale bar of A and B is 1 mm for both. (**E**) Outfitting the light-guided cyborg beetle by mounting an electronic backpack onto the pronotum of the beetle. (**F**) The body-steering angles of beetles controlled by the light source on the backpack. (**G**) The turning velocity of cyborg beetles. *** indicates an extremely significant difference (*p* < 0.001). ** indicates a high significant difference (*p* < 0.01). n.s. indicates no significant difference (*p* > 0.05). (**H**) Potential applications of light-controlled cyborg beetles in future research.

We developed a remotely controllable electronic backpack for locomotion control (attraction) using blue LEDs. The control system of the electronic backpack employs a commercial Arduino pro mini microcontroller development board, which includes an ATMEGA 328P chip (Microchip, Chandler, AZ, America) for storing and running custom programs, receiving radio signals, and controlling the attractive light switches (Figure 4A). This chip also provides up to 14 digital I/O pins (6 of which offer PWM output) and 6 analog input pins, offering significant potential for expanding the backpack’s functionality. We soldered two blue LEDs (Figure 4A) onto the A1 and A3 pins at the center of the chip, providing left and right attraction light sources, respectively (Figure 4D). To achieve remote control, we selected a commercial radio-receiving module to receive control signals. This module uses the SYN480R chip (Synoxo, Paris, France) paired with a 6.7458 MHz crystal oscillator to receive 433 MHz radio-frequency signals (Figure 4A). Correspondingly, the operating terminal is constructed by connecting a 433 MHz radio-frequency-transmitting module equipped with an F115 chip and a 13.560 MHz crystal oscillator to another microcontroller development board. By detecting the button pressed by the user, different control signals are sent to achieve manually induced light-guided directed locomotion of insects.

Only a small portion of the light emitted by the LED is directly received by the compound eyes of *E. florensis*. By consulting the parameters related to spatial distribution in the LED component manual (https://item.szlcsc.com/datasheet/TJ-L234FGHTCGLFLC6B-A5/439847.html?spm=sc.gb.xds.a___sc.hm.hd.ss&lcsc_vid=EVReUQcFQllZBVdRR1RfU1RVTgBWBlIHEVVeU1AERgQxVlNSR1BdVFFSQFVWVztW, accessed on 2 January 2025), it can be seen that the light distribution angle (Figure 4E) has an approximately quadratic relationship with relative light intensity, satisfying the following nonlinear fitting relationship:

(1) $$y=a\theta^{2}+b\theta +c$$

where **y** represents the relative light intensity, which is the ratio of the emitted light intensity to the maximum light intensity and ranges from 0 to 1.0. **θ** represents the light distribution angle. After calculation, the coefficients are determined as **a** = −4.17×10^−5^, **b** = −7.52×10^−3^, **c** = 0.98, with a coefficient of determination **R^2^** = 0.98. The calculation of coefficients **a**, **b**, and **c**, as well as **R^2^**, typically involves fitting the experimental data to the quadratic function (1) using the curve_fit method from the scipy.optimize package in Python. The value of **R^2^** indicates the goodness of fit, where **R^2^** = 0.98 suggests a strong fit between the model and the data.

Based on the spatial distribution characteristics of the LED, it is known that the light intensity proportion is the same for light radiation distributed at the same angle. If the LED is perpendicular to the illuminated plane, then the light intensity is uniform across a circle with a radius denoted as:

(2) r=d tanθ

where **d** is the distance between the LED and the illuminated plane (Figure 4E). Alternatively, this relationship can be expressed as:

(3) $$\theta =arctan\frac{r}{d}$$

Using the fitting relationship described in Equation (1), the total light radiant flux of the LED can be calculated as follows:

(4) $$L=\int_{r_{min}}^{r_{max}}2\pi r(a{\;arctan}^{2}\frac{r}{d}+b\;arctan\frac{r}{d}+c)\;dr$$

As previously mentioned, the light intensity is uniform across a circle with a given radius of radiation. The compound eyes of insects are distributed within a ring-shaped area within the range of light radiation. Let the lower angle limit of this ring area be **θ_a_** and the upper angle limit be **θ_b_**. The proportion of light radiation within this ring area can be calculated as follows:

(5) $$\omega_{r}=\frac{\int_{r_{a}}^{r_{b}}2\pi r(a\;{arctan}^{2}\frac{r}{d}+b\;arctan\frac{r}{d}+c)dr}{\int_{r_{min}}^{r_{max}}2\pi r(a\;{arctan}^{2}\frac{r}{d}+b\;arctan\frac{r}{d}+c)dr}$$

Based on Equation (2), the area of the ring can be calculated as follows:

(6) $$S_{r}=\pi d^{2}({tan}^{2}\theta_{b}-{tan}^{2}\theta_{a})$$

Based on the area ratio, the proportion of light radiation received by the insect’s compound eyes, denoted as **ω_e_**, can be calculated as:

(7) $$\omega_{e}=\frac{S_{e}\omega_{r}}{\pi d^{2}({tan}^{2}\theta_{b}-{tan}^{2}\theta_{a})}$$

The optical power received by the beetle’s compound eyes, denoted as **P_e_**, can be expressed as:

(8) $$P_{e}={\rho P}_{l}\omega_{e}$$

where **P_l_** is the power of the LED, and **ρ** is the luminous efficiency, which in this model takes an empirical value of 40%. In this electronic backpack, the current-limiting resistor **R_cl_** is 4.7 kΩ. The nominal voltage of the power supply is 3.7 V, and the measured voltage at full charge is between 4.1 and 4.2 V, with an average operating voltage **U_t_** taken as 4.0 V. Considering that the blue LED itself has a voltage drop (**U_ld_**) of approximately 1.7 V, according to Ohm’s law, we have:

(9) $$P_{l}=\frac{U_{ld}(U_{t}-U_{ld})}{R_{cl}}$$

Through calculation, it can be determined that the power **P_l_** of the LED is approximately 0.83 mW.

Based on Equation (1), it can be calculated that when the light distribution angle **θ** is 87.74°, the relative light intensity is 0, indicating that the maximum radiation angle **θ_max_** of the LED is approximately 87.74°. Through measurement, the vertical distance **d** between the LED and the plane where the compound eyes are located is 6.54 mm, at which point **r_max_** = 165.72 mm. Correspondingly, **θ_min_** is 0°, i.e., **r_min_** = 0 mm. The measured values of **θ_a_** and **θ_b_** are 56.06° and 66.91°, respectively (N = 3), with corresponding **r_a_** and **r_b_** of 9.72 mm and 15.34 mm; the measured area of the compound eyes is 3.79 mm^2^ (N = 3). According to Equations (5) and (7), the values of **ω_r_** and **ω_e_** are 5.96% and 0.51 ‰, respectively. In summary, the optical power received by a single compound eye of the beetle is approximately 0.169 μW.

**Figure 5:**
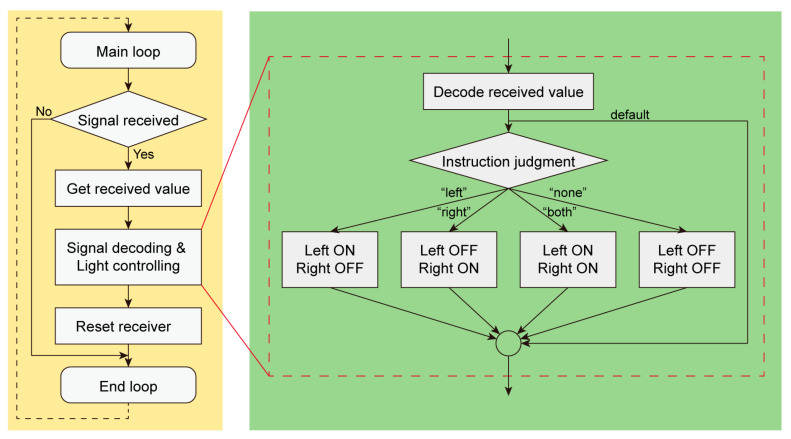
A flow diagram of the electronic backpack of light-guided cyborg beetle.

According to the results of behavioral experiments, we determined that female *E. florensis* exhibit slightly higher activity levels compared to males; thus, we utilized females as the carriers for the electronic backpack in our insect–machine hybrid system (Figure 4C,D). The working logic of the electronic backpack is shown in Figure 5. We conducted steering control tests with eleven female adults. The results indicated that the blue LEDs could induce *E. florensis* to carry out same-direction turning (Video S1). After activating the attraction light sources on the electronic backpack, the body axes of the tested individuals deflected by 62.34° and 58.35° to the left and right sides, respectively, within 5 s (Figure 4F). Before activating the light sources, the average turning angular velocities of the left-turn and right-turn test groups were 0.70° ± 2.32° and 1.79° ± 1.31° (AVE ± SE), respectively, with no significant difference (*p* > 0.05). The average turning angle was 12.47° ± 2.63° after activating the left light source, which was significantly higher than before turning on the light (*p* < 0.01). The angular velocity for right-turn control was −11.67° ± 1.35°, showing a extremely significant difference compared to before turning on the light (*p* < 0.001) (Figure 4G).

## 4. Discussion

The results demonstrate that blue light can effectively guide the steering motion of the beetle. In the following discussion, we explore the implications of these fundings and their potential applications. For the first time, we have achieved a repeatable and precise guidance system for light-guided walking insects, coupled with a wirelessly controllable optoelectronic device. This study introduces a novel approach to controlling insect locomotion, specifically for turning behavior. Through ERG and phototactic behavioral studies of *E. florensis*, blue LEDs with wavelengths between 463 and 469 nm were identified as the optimal guiding light source. A light-guided electronic backpack was constructed, and experiments with the insect–machine hybrid system were conducted to verify the feasibility of controlling locomotion through the exploitation of insect phototaxis.

The electrophysiology of compound eyes reflects an insect’s sensitivity to various wavelengths of light. Insects are highly diverse, and their sensitivity to different wavelengths varies by species, influenced by the types of opsins in their compound eyes. Most insects are sensitive to three main wavelengths—green, blue, and ultraviolet—corresponding to long-wavelength opsins, short-wavelength opsins, and ultraviolet opsins, respectively [19,22]. Some species also exhibit sensitivity to red and yellow light [18]. In this study, we measured the photosensitivity of *E. florensis* using four common visible-light wavelengths. The results showed that *E. florensis* exhibited the strongest ERG responses to blue and green light, weaker responses to yellow, and significantly lower responses to red light. Based on these findings, we conclude that *E. florensis* can detect blue, green, and yellow but has reduced sensitivity to red. Additionally, there were no significant differences in sensitivity between males and female beetles.

It is important to distinguish between light sensitivity and phototaxis in insects. Sensitivity to light refers to an insect’s ability to detect specific wavelengths, while phototaxis reflects the insect’s preference or behavior in response to specific light sources. Phototactic responses can vary greatly across species and are often related to feeding and reproductive behaviors. For instance, pollen beetles Meligethes aeneus [23] and oriental fruit flies Bactrocera dorsalis [24], which feed on flowers and fruits, use yellow light to locate host plants. In contrast, insects that feed on plant tissue, such as the tobacco cutworm Spodoptera litura [25], the oriental armyworm Grapholita molesta [15], and the diamondback moth Plutella xylostella [26], often exhibit positive phototaxis towards green light around 520 nm. Our research shows that while *E. florensis* has strong light sensitivity to blue, green, and yellow light, its behavioral responses differ significantly. Under blue light, both males and females exhibited strong positive phototaxis, with clear directional movement toward the light. However, under yellow light, both sexes spent no time in the illuminated area, and their movement patterns were more scattered, indicating negative phototaxis. Under green and red light, no significant preference was observed, suggesting a lack of phototactic selectivity toward these wavelengths. We interpret the lack of selectivity toward green light as a behavioral trait, while the reduced sensitivity to red light is likely due to physiological factors.

Notably, this study employed a square behavioral chamber. Due to the walls of the chamber, beetles often crawled along the acrylic walls after reaching the chamber’s edge, eventually reaching the corners. If they did not perform large-angle turns, they remained in the corners of the chamber. Thus, under yellow light, many trajectories were found in the areas farthest from the light source (between −135° to −165° and −15° to −45°), rather than in the opposite direction (−75° to −105°). In contrast, under blue light, the majority of beetles’ trajectories were concentrated within 75° to 105°, accurately reflecting their strong phototactic response to blue light.

In terms of overall activity, females exhibited slightly higher activity level than males, particularly under green and red light, where females spent more time active than males. However, under blue and yellow light, both sexes showed similar levels of activity. We propose that positive and negative phototaxis drive insect movement, which is likely physiological in nature, although the exact mechanism remains to be explored. When confronted with green or red light, which the beetles neither prefer nor can perceive, their movement is less driven, and the proportion of time spent active reflects their intrinsic activity levels.

We also attempted to assess activity by measuring movement speed and distance, presented in Figure 1D. However, due to the physical barriers of the acrylic chamber walls, the measured speed values and distributions were lower than theoretical expectations. While parameters such as real-time speed, average speed, and movement distance can be calculated through video analysis, we consider these values to be approximate and recommend larger, open testing environments for more accurate measurements.

Numerous studies have proposed methods for controlling insect locomotion, particularly using electrical stimulation. These methods cover a wide range of species, including beetles [3,4,27], cockroaches [2,28,29], locusts [5,30,31], and moths [32,33], as well as various movement types, such as walking [2,27,29], flying [3,32,33], and jumping [5,30,31]. Studies have also proposed control types such as turning control [3,5,29], motion initiation [3,5] and cessation [3]; stimulation sites including antennae [8,29], compound eyes [34], thorax [3,33], legs [4,5], and cerci [29]; and stimulated tissues including nerves [28,29], optic lobes [34], and muscles [3,4,5]. Except for a few studies [3,4], most electrical stimulation protocols exploit evasive behaviors, forcing insects to move away from the stimulus [2,3,5]. These methods, while stable and versatile, are invasive, often requiring electrode implantation, which can cause irreversible damage to insects, such as drilling holes or amputating antennae. Some studies claim these procedures do not significantly affect insect lifespan, but physical damage remains a concern [8]. In contrast, light-guided directional movement provides a non-invasive method for controlling insect locomotion. This technique uses the insect’s natural phototaxis to induce directional changes, without the need for surgical intervention. Since it relies solely on light, it avoids the negative physiological impacts associated with electrical stimulation.

Some studies have explored non-invasive electrode implantation methods, such as early-metamorphosis insertion technology (EMIT) [6,7] and non-invasive conformal electrodes [8]. EMIT leverages the resorption and reconstruction of tissues during the pupal stage of holometabolous insects, allowing for stable electrode integration as the insect develops. However, this technique requires invasive procedures during the pupal stage and is limited to holometabolous species, such as moths. In contrast, non-invasive conformal electrodes can be fitted around the antennae of insects, but this approach is still limited to species with suitable antenna structures and remains complex and slow. In contrast, our light-guided electronic backpacks can be easily attached to the back of the insect, offering a simpler, faster, and less invasive solution.

Previous attempts at light-guided insect locomotion control, such as the 2008 study on the beetle Cotinis texana, did not achieve the desired results due to inadequate light wavelength or intensity [35]. Our approach differs in several key aspects, including the use of blue LEDs based on comprehensive biological testing, fewer light sources for reduced power consumption, and the ability to provide remote control. Furthermore, we adjusted the LED brightness to avoid triggering negative phototaxis, a feature not accounted for in previous studies. However, light-guided locomotion remains sensitive to environmental lighting conditions. The tests in this study were conducted under fluorescent lighting with low illuminance, which allowed light-guided steering to function effectively. In contrast, intense environmental lighting, such as direct sunlight, could interfere with the light source and reduce steering efficiency. Additionally, while the current system is effective for walking tasks, challenges remain in guiding flight motion, particularly in terms of pitch control. Therefore, the cyborg beetle system described in this study is particularly suitable for executing walking tasks in dark environments, such as rubble rescue operations and cave ecological monitoring.

Finally, while *E. florensis* is a nocturnal species, limiting its potential for daylight tasks, previous studies have demonstrated methods for enhancing insect activity through both chemical and physical means. For instance, chemicals like methyl salicylate can be used to activate cockroaches’ locomotor activity [35]. Further exploration is needed to identify substances that could enhance the locomotor ability of *E. florensis*, and adjusting the insect’s circadian rhythm may offer a way to expand its operational window to any time of day.

In conclusion, this study presents a significant advancement in the field of insect locomotion control, providing new insights into the potential applications of light-guided cyborg insects for tasks such as search and rescue and ecological monitoring.

## Data Availability

The datasets presented in this article are not readily available because of technical limitations. Requests to access the datasets should be directed to the corresponding author.

## Supplementary Materials

The following supporting information can be downloaded at: https://www.mdpi.com/article/10.3390/biomimetics10080513/s1, Video S1: Steering control of beetle *Endebius florensis*.
